# A case of anterior persistent hyperplastic primary vitreous associated with morning glory disc anomaly and retinopathy of prematurity like retinopathy in a term-born child

**DOI:** 10.1186/s12886-021-02200-1

**Published:** 2021-12-28

**Authors:** Hao Zhang, Kaiqin She, Fang Lu

**Affiliations:** Department of Ophthalmology, West China Hospital, Sichuan University, No.37, Guoxue xiang, Chengdu, 610041 Sichuan China

**Keywords:** MGDA, PHPV, ROP-like retinopathy

## Abstract

**Background:**

Association of morning glory disc anomaly (MGDA) with persistent hyperplastic primary vitreous (PHPV) has been reported earlier. Retinopathy of prematurity (ROP) like retinopathy in preterm babies with optic disc anomalies has also been published. Our case is unique in terms of presence MGDA, PHPV, unilateral ROP like retinopathy in a term infant with normal birth weight.

**Case presentation:**

A 5-month-old girl, born at term with a birth weight of 3750 g, presented with anterior PHPV, MGDA and ROP like retinopathy. In order to prevent retinal detachment, she received 360 degree barrage laser photocoagulation at the edge of the optic disc excavation of the left eye. In the follow-up a month later, laser scars were found in her left fundus without other complications.

**Conclusion:**

PHPV and MGDA with ROP like retinopathy in term and normal weight baby is rare. The peripheral avascular retinal area, caused by the dragging of the defected optic disc, might have been more vulnerable to the oxygen change after birth which resulted in ROP like retinopathy. High sensitivity to oxygen results in a series of changes such as upregulation of VEGF and IGF-1 may cause ROP-like retinopathy.

## Background

Morning glory disc anomaly (MGDA) is a rare congenital optic disc malformation characterized by funnel shaped excavation of the dysplastic optic disc, covered with glial tissue, increasing number of straight radiating vessels from the disc margin, peripapillary pigmentation and excavation with or without macular capture [1]. Magnetic resonance imaging(MRI) shows unilateral abnormal tissue associated with distal intra-orbital part of optic nerve, discontinuous uveo-scleral coat and effacement of subarachnoid space [2].

Other congenital anomalies associated with MGDA are cataract, posterior lenticonus, lens coloboma, persistent hyperplastic primary vitreous (PHPV), retinal detachment and secondary glaucoma [3–5]. Among these, the combined incidence of PHPV has been reported to be as high as 25.88% [5]. PHPV, a congenital developmental disorder of the eye, is the result of the failure of the primary vitreous and the hyaloid vasculature to regress. The high incidence of MGDA with PHPV implicates that there may be genetic or embryonic links in the pathogenesis of these two conditions [5, 6].

Retinopathy of prematurity is a potentially blinding condition associated with abnormal vasculature in response to abnormal angiogenesis in preterm and low birth weight babies. The pathogenesis of ROP has two phases: the first phase involves delayed physiologic retinal vascular development, and the second phase involves vasoproliferation [7, 8]. Vascular endothelial growth factor (VEGF) plays a major role in its pathogenesis. In the first phase, the hyperoxia after the delivery (atmospheric oxygen and the supplement oxygen) causes suppression of vascular endothelial growth factor (VEGF) and other angiogenic factors. In the subsequent phase, the peripheral avascular retina becomes ischemic, which triggers the release of angiogenic factors such as VEGF, resulting in neovascularization. Low birth weight and prematurity are the most important risk factors of ROP.

## Case presentation

**Fig. 1:**
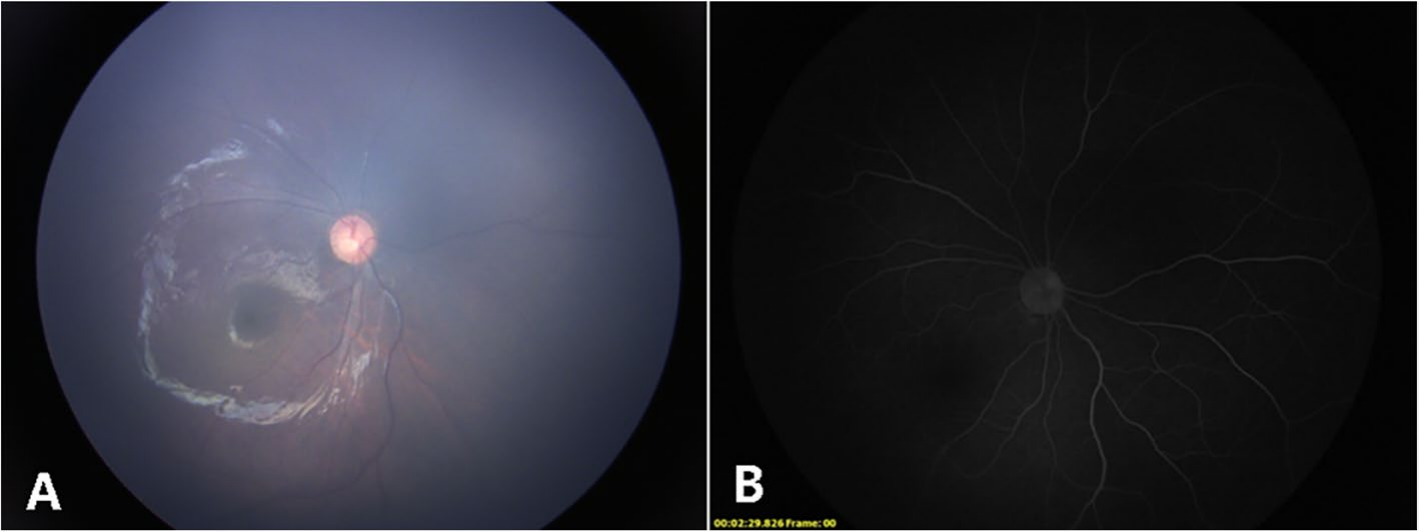
Title, Photographs of the fundus of the patient’s right eye. Legends, The colour fundus photograph of the right eye (**A**) showing normal optic disc and retina, corresponding FFA (**B**) also showing normal vessels architecture without any leakage

**Fig. 2:**
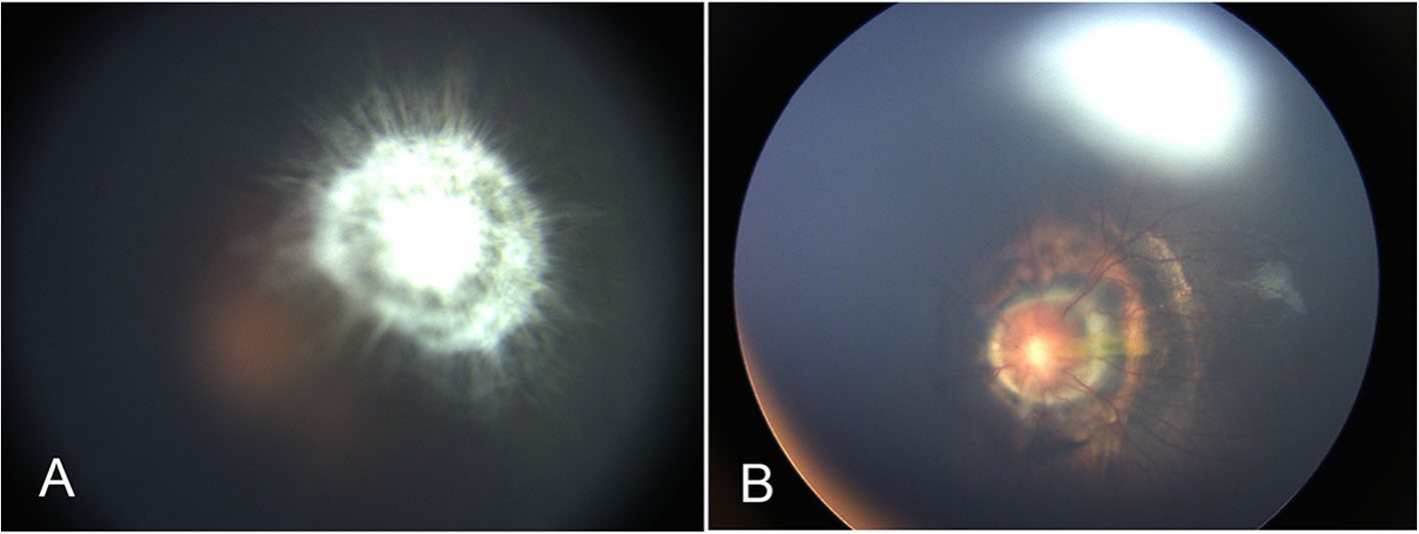
Title, Photographs of the fundus of the patient’s left eye. Legends, A whitish star shaped mass attached to the posterior lens capsule (**A**); enlarged and excavated optic disc with macular dragging to temporal side (**B**)

**Fig. 3:**
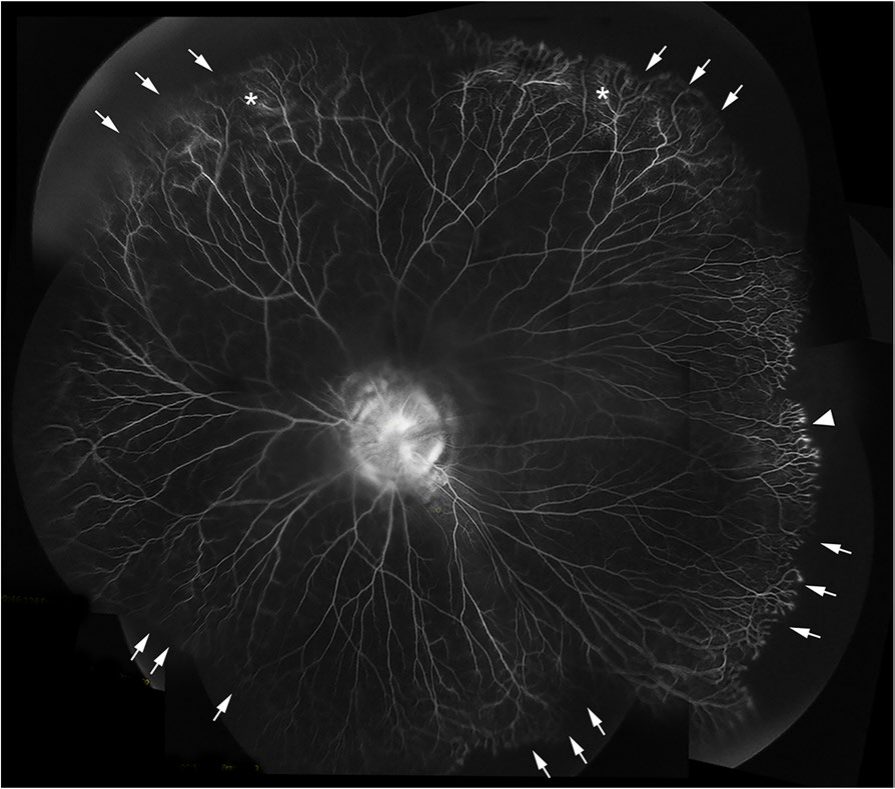
Title, Montage fluorescein angiogram of the patient’s left eye. Legends, Montage FFA of the baby’s left eye showing enlarged hyperfluoroscence optic disc with multiple radiating vessels from the optic disc, abnormal vascular branching, bulbous ending of the vessels (white arrow heads) with leakage at the periphery (white stars), 360-degree avascular retinal periphery with well-defined vascular and avascular border (white arrows)

**Fig. 4:**
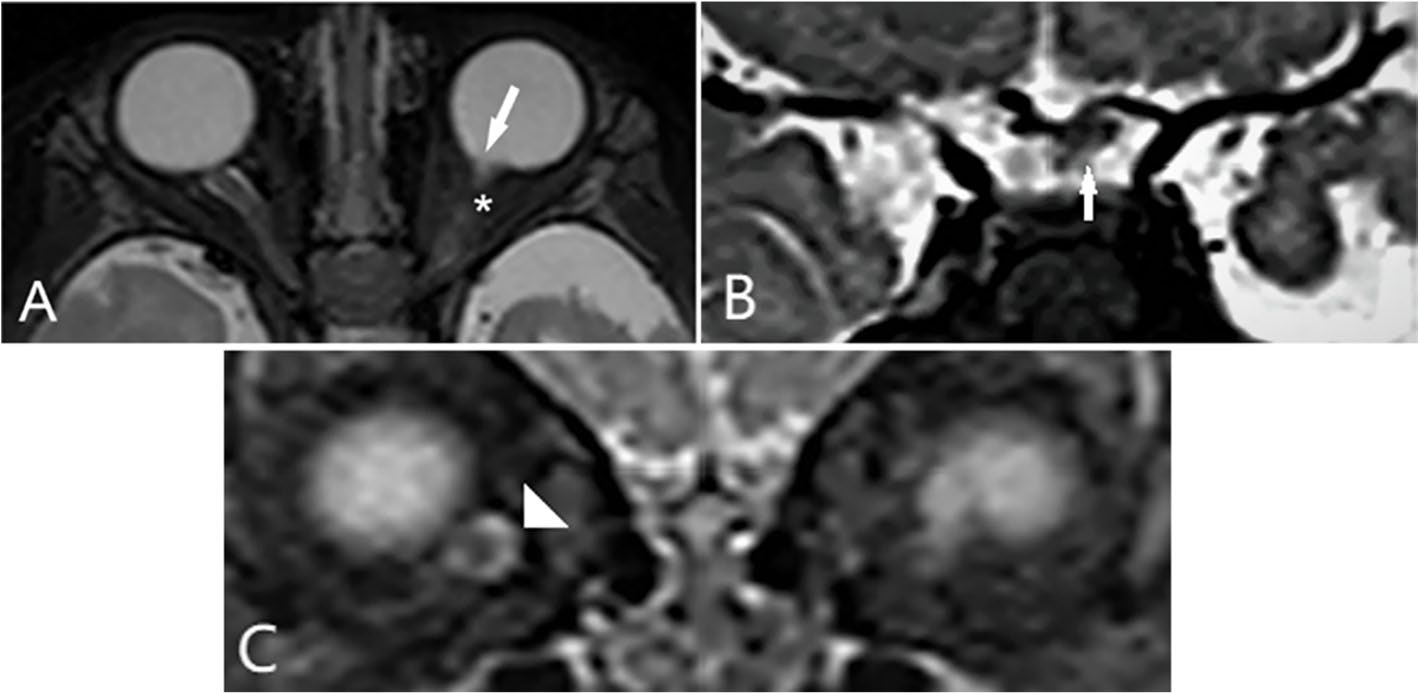
Title, MRI of the orbit. Legends, Axial T2WI through the orbits of the patient demonstrated (**A**) excavated optic nerve head with scleral defect (white arrow) and tortuous optic nerve (white star). The coronal T2W1 image (**B** and **C**) showing asymmetry of optic chiasma with distortion in left side (white arrow); right optic nerve surrounded by enhancing CSF (white arrow head) which is absent in left side

**Fig. 5:**
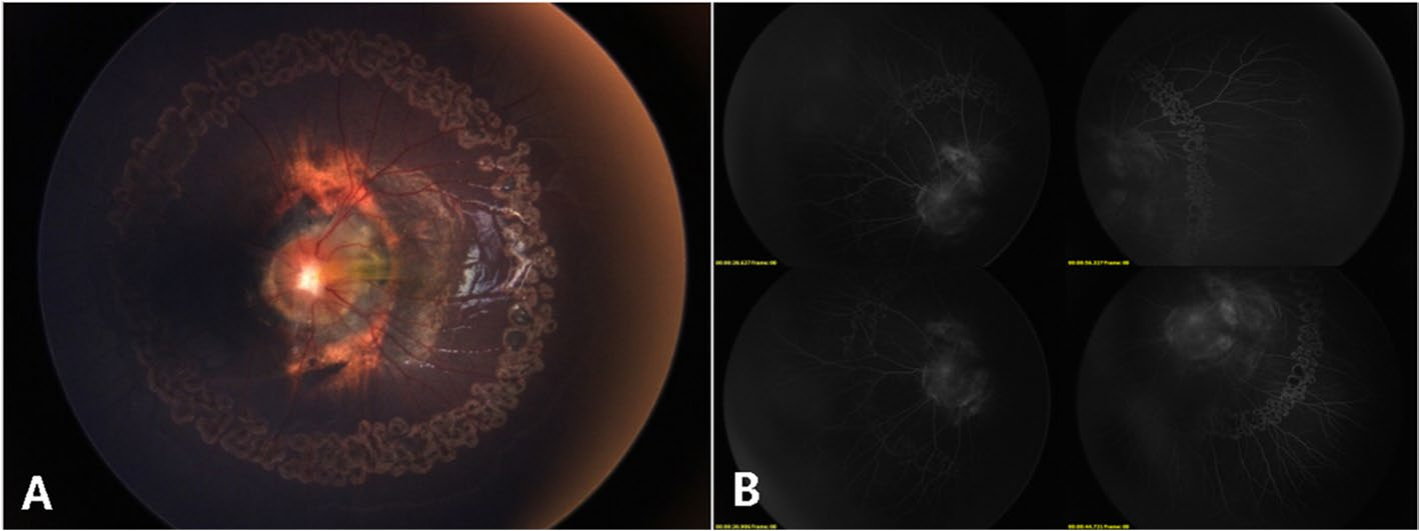
Title, Fundus photograph and fluorescein angiogram of the left eye at last follow up visit. Legends, Left eye color fundus photograph showing 360-degree barrage laser surrounding the large excavated optic disc with multiple radiating vessels (**A**). Corresponding FFA shows staining of laser scars (**B**)

A 5-month-old girl, born at term with a birth weight of 3750 g, was presented to our outpatient department with the chief complaint of deviation of both eyes to the left side by the parents. The baby had history of oxygen therapy in neonatal intensive care unit (NICU) for neonatal pneumonia for three days after birth, there was no other systemic abnormalities. The exact duration and mode of oxygen inhalation was not known. Except for the deviation of both eyes to the left, there was no other abnormal facial features of the baby. A preliminary torch light examination was done at the time of admission later we examined the child under anesthesia under an operating ophthalmic microscope. The anterior segment of the right eye and fundus were within normal limits (Fig. 1). The left eye cornea and lens were transparent, there was a whitish star shaped tissue close to posterior lens capsule (Fig. 2A). Vitreous cavity was anechoic. Fundoscopy revealed attached retina, a large excavated dysplastic disc with multiple vessels radiating from the disc margin. The peripapillary area showed chorio-retinal pigmentary changes. The macula was dragged to the temporal side. (Fig. 2B). Fluorescein angiography showed multiple radiating retinal vessels from the disc to periphery. The disc and its surrounding annular area showed hyper fluorescence without leakage (Fig. 3). The retinal vessels showed abnormal branching with bulbous terminals and leakage of fluorescein dye at the periphery. The peripheral retina showed 360 degree non perfusion with a defined border between vascular and avascular retina (Fig. 3). The B-scan ultrasonography of the left eye revealed disc excavation. MRI brain and orbit showed no obvious abnormality in brain parenchyma, normal right orbit, and right optic nerve with surrounding ring of enhancing cerebrospinal fluid (CSF). But in the left eye, the optic nerve head was excavated with scleral defect (Fig. 4A). The intra-orbital part of optic nerve in left side was tortuous-thickened and this was extending up to the chiasma (Fig. 4B). The absence of CSF enhancement surrounding the left optic nerve revealed abnormal development of the optic nerve sheath (Fig. 4C). The child received barrage laser photocoagulation to the edge of optic disc excavation of left eye to prevent retinal detachment due to dysplastic disc. In the follow-up visit after 1 week, laser scar surrounding the disc could be appreciated in fundoscopy. At last follow up after 9 months of laser intervention, the left eye fundus was stable (Fig. 5).

## Discussion and conclusions

ROP-like retinopathy has been reported in full-term infants with low birth weight, multiple births, respiratory distress syndrome (RDS), phototherapy, and oxygen administration [9]. But all these cases had bilateral disease, while our patient presented ROP-like retinopathy only in the left eye, which had MGDA and PHPV. Asymmetric ROP has also been reported in premature babies associated with optic disc anomalies, including peripapillary staphyloma and optic nerve hypoplasia [10, 11], however our case is unique in presence of unilateral ROP like retinopathy without prematurity and low birth weight though history of NICU admission and oxygen therapy were present. Familial exudative vitreoretinopathy (FEVR), a bilateral, hereditary condition, is the commonest cause of avascular retina in full term babies. In our case, the right eye vasculature was within normal limits with mature retina, so FEVR was excluded from the diagnosis. The etiology of this condition remained uncertain as no genetic analysis was done, though there was a possibility of environmental influence related to NICU stay and oxygen administration.

MGDA associated with peripheral retinal nonperfusion has been reported and well recognized. The peripheral avascular retina attributed by the anomaly of defected optic disc is the character of MGDA [10, 12, 13], which is exactly the same showed in our case. The endings of the peripheral retinal vessels at the junction between the retinal perfusion and retinal non-perfusion zone in MGDA presented as increasing in numbers of branches with fine tips without fluorescein leakage, just like “brushfire “. However, in this case, the differences on the terminals of these brushfire vessels are dilating tips along with gentle fluorescein leakage, this is quite similar to those vascular tips presented in ROP. Our hypothesis is that the presetting peripheral avascular areas induced by the dragging of the defected optic disc, are more vulnerable to the oxygen fluctuations after birth which resulted in series of changes such as down and upregulation of VEGF thus forming ROP-like retinopathy [14]. We suggest a thorough fundoscopy examination including periphery of the retina and fundus fluorescein angiogram should be performed in patients with MGDA, and the peripheral avascular retina should be followed regularly.

## Data Availability

All data analysed during this study are included in this published article.

## Abbreviations

MGDA: Morning glory disc anomaly
PHPV: Persistent hyperplastic primary vitreous
ROP: Retinopathy of prematurity
FEVR: Familial exudative vitreoretinopathy
RDS: Respiratory distress syndrome
VEGF: Vascular endothelial growth factor
IGF-1: insulin-like growth factor-1
